# Oxidative Phosphorylation in Uncoupled Mitochondria

**DOI:** 10.1002/bies.70038

**Published:** 2025-07-06

**Authors:** Henver S. Brunetta, Marcelo A. Mori, Alexander Bartelt

**Affiliations:** ^1^ Department of Cellular and Molecular Biology Karolinska Institutet Stockholm Sweden; ^2^ Department of Biochemistry and Tissue Biology University of Campinas Campinas Brazil; ^3^ Chair of Translational Nutritional Medicine, TUM School of Life Sciences, Research Department of Molecular Life Sciences Technical University of Munich Freising Germany; ^4^ Else Kröner Fresenius Center for Nutritional Medicine Technical University of Munich Munich Germany; ^5^ Institute For Cardiovascular Prevention (IPEK), Faculty of Medicine Ludwig‐Maximilians‐Universität München Munich Germany; ^6^ Institute For Diabetes and Cancer (IDC), Helmholtz Center Munich German Research Center For Environmental Health Neuherberg Germany; ^7^ German Center for Cardiovascular Research, Partner Site Munich Heart Alliance Ludwig‐Maximilians‐University Hospital Munich Germany; ^8^ German Center for Diabetes Research Neuherberg Germany

**Keywords:** adipocytes, bioenergetics, metabolism, mitochondria, obesity, thermogenesis, UCP1

## Abstract

Mitochondrial membrane potential is highly dependent on coupled as well as uncoupled respiration. While brown adipose tissue (BAT) mediates non‐shivering thermogenesis (NST), a highly adaptive bioenergetic process critical for energy metabolism, the relationship of coupled and uncoupled respiration in thermogenic adipocytes remains complicated. Uncoupling protein 1 (UCP1)‐mediated proton leak is the primary driver of NST, but recent studies have shown that oxidative phosphorylation may be an underappreciated contributor to UCP1‐dependent NST. Here, we highlight the role of ATP synthase for BAT thermogenesis and discuss the implications of fine‐tuning adrenergic signaling in brown adipocytes by the protein inhibitory factor 1 (IF1). We conclude by hypothesizing future directions for mitochondrial research, such as investigating the potential role of IF1 for mitochondrial substrate preference, structural dynamics, as well as its role in cell fate decision and differentiation.

## Graphical abstract

Brown adipose tissue (BAT) mediates non‐shivering thermogenesis (NST) through activation of uncoupling protein 1 (UCP1). Due to the uncoupled nature of BAT, oxidative phosphorylation is an underappreciated contributor to NST. Here, we discuss the implications of Inhibitory Factor 1 modulation, an endogenous inhibitor of ATP synthase, for BAT physiology.

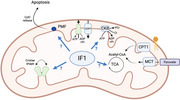

## 1 Introduction

**FIGURE 1:**
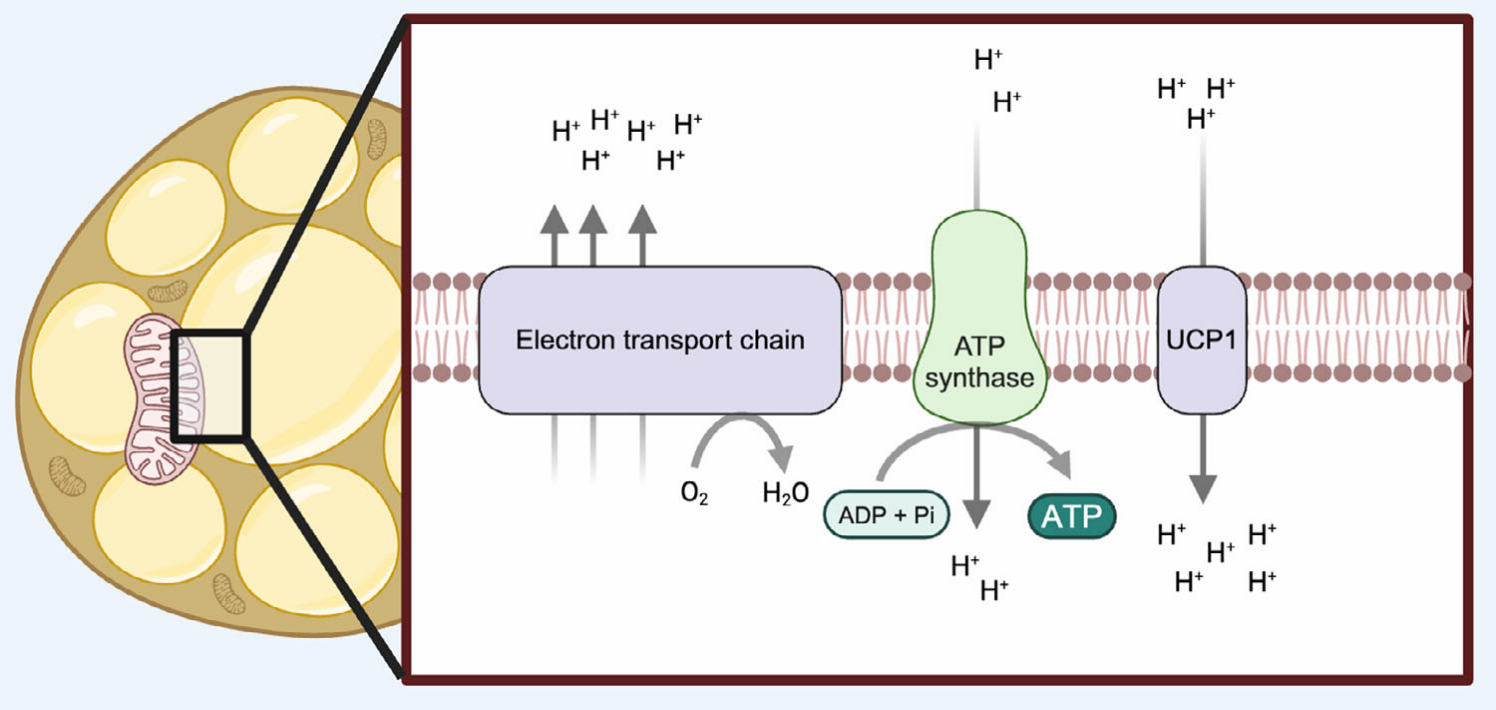
UCP1 activity in brown adipocytes modulates oxygen consumption. Upon adrenergic stimulation, UCP1 increases its conductance, allowing the vectorial flow of protons from the intermembrane space into mitochondrial matrix, resulting in mitochondrial depolarization and increase in oxygen consumption [1, 4, 5].

Non‐shivering thermogenesis (NTS) is an adaptive mechanism by which the body generates heat to maintain core temperature in a homeostatic physiological range [1, 2, 3]. In mammals, brown adipose tissue (BAT) has the unique ability to generate heat through NST, which involves a controlled process of uncoupling protein 1 (UCP1) activation in the inner mitochondrial membrane of brown adipocytes [4, 5]. After the identification of BAT in humans [6, 7], several research groups around the world searched for means to leverage the metabolic capacity of BAT to dissipate high caloric nutrients through uncoupling respiration to treat obesity, a chronic condition of positive energy imbalance [8]. Pharmacological uncouplers (i.e., FCCP or DNP) have been proposed to combat obesity, however, the non‐selective nature of such agents offers a high risk for health alongside a small therapeutic window. In this context, activation of already existing endogenous uncouplers, such as UCP1 in adipocytes, has been suggested. Mechanistically, UCP1 activation upon adrenergic signaling allows the movement of protons from the intermembrane space into the mitochondrial matrix [5, 9]. This vectorial flow of protons is rapidly matched by an increase in electron transport chain (ETC) activity, substrate oxidation, and cellular oxygen consumption (Figure 1). In rodents and humans, activation of BAT with β‐adrenergic agonists increases whole‐body oxygen consumption, highlighting the capacity of BAT to modulate energy expenditure [10, 11, 12, 13].

Peter Mitchell's chemiosmotic theory conciliated the activity of ETC with oxidative phosphorylation (OxPhos) by stating that the protonmotive force (PMF) generated by mitochondrial respiration drives protons back into the mitochondrial matrix through the F_1_‐F_O_‐ATP synthase (hereafter called ATP synthase), providing energy for ATP production from ADP and phosphate [14]. In BAT, the contribution of OxPhos to total oxygen consumption has been considered minor to NST for several years. This argument was supported by two main findings: First, brown adipocytes in BAT display relatively low content of subunit c of ATP synthase [15], therefore, rendering a virtually absent complex V activity [16]. Second, due to dynamic lipid mobilization in BAT upon activation, baseline mitochondrial membrane potential (MMP) would be below the threshold of complex V‐mediated ATP synthesis [17]. Challenging this view, researchers have dug deeper into the control of mitochondrial bioenergetics in BAT using the advances of new techniques. For example, Shirihai's group identified two mitochondrial populations residing in brown adipocytes with distinct bioenergetics features, in which lipid droplet‐associated mitochondria retain the capacity to produce ATP even under adrenergic stimulation [18]. More recently, it was identified that energetic stress in fibroblasts divides mitochondria into two subpopulations: One of them being devoid of mitochondrial cristae and ATP synthase, rendering an organelle specialized in redox potential control with no contribution to ATP production, whereas the other population displays canonical mitochondrial features, such as cristae shape and ATP synthase [19]. Finally, mitochondrial respiration in brown adipocytes is reduced in the presence of oligomycin, an ATP synthase inhibitor [20]. These findings highlight the role of oxidative phosphorylation by ATP synthase in BAT. Here we discuss the role of inhibitory factor 1 (IF1), a small protein natural inhibitor of ATP synthase, and its biological consequences for UCP1‐dependent and independent thermogenesis.

## 2 Inhibitory Factor 1 and the Control of ATP Synthase Function

ATP synthase, the enzyme responsible for ATP synthesis in the OxPhos system, is a proton‐translocating ATPase, normally operating as an ATP synthase driven by MMP generated by ETC activity. The directionality of the enzyme is dictated by the balance between free energy availability (i.e., f[ADP]) and electrochemical gradient [21]. However, it is now recognized that under certain conditions, such as low MMP or mitochondrial dysfunction, the reverse mode of ATP synthase is potentiated, generating MMP at the cost of mitochondrial ATP consumption [22, 23]. In 1963, Pullman and Monroy discovered the small mitochondrial protein IF1, encoded by *Atp5if1* gene, located in the mitochondrial matrix. IF1 is activated when mitochondrial matrix pH is low, resulting in the inhibition of ATP synthase hydrolytic activity when operating in the reverse mode [24]. The mammalian IF1 protein contains 106–109 amino acids (depending on the species of origin) with the first 25 amino acids representing a mitochondrial targeting sequence that is cleaved off once within the mitochondria to form the functionally mature IF1 protein of 84 amino acids [25]. The physical interaction of IF1 with ATP synthase is hypothesized to prevent cellular ATP depletion by mitochondria [26].

The large capacity of mitochondria to consume cytosolic ATP when ATP synthase is working in the reverse mode has been demonstrated by independent groups in various contexts, such as upon mitochondrial mutations or chemically induced mitochondrial depolarization [23, 27]. However, experiments on isolated mitochondria demonstrated that when the ETC is compromised, ATPase reverses and MMP is maintained for as long as matrix substrate‐level phosphorylation is functional [22]. This finding raises the question whether mitochondria consume cytosolic ATP when hydrolase activity is high. While the cellular source of ATP as a substrate for ATP synthase during the reverse mode remains debatable, modulation of IF1 levels offers an additional regulator of ATP synthase that goes beyond MMP and matrix _f_[ADP] [28, 29].

**FIGURE 2:**
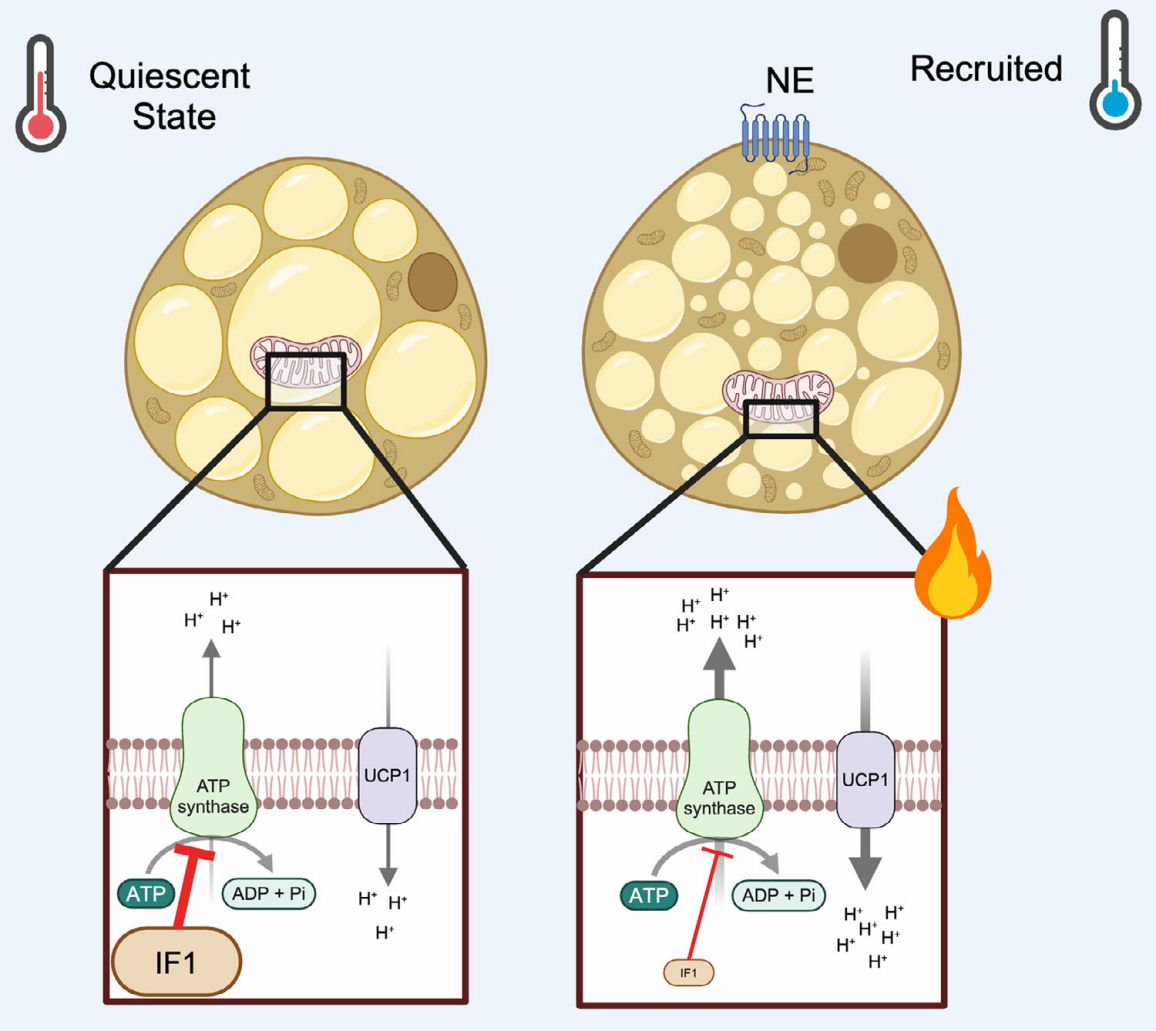
IF1 is downregulated upon cold exposure to facilitate the reverse mode of ATP synthase in brown adipocytes. Reduction of IF1 levels in BAT prevents excessive mitochondrial depolarization once UCP1 is activated. Downregulation of IF1 upon cold exposure seems to be a necessary event for BAT recruitment, given its overexpression impairs adrenergic‐stimulated oxygen consumption and overall mitochondrial respiratory capacity [13].

It has been shown that IF1 regulates the forward activity of ATP synthase in different cells and tissues [30, 31]. Structural work showed, however, that IF1 physically binds to the F_1_‐catalytic domain of the ATP synthase, resulting in an inhibition of its hydrolytic activity with no apparent regulation of its synthetic activity [21, 32]. The physiology of thermogenesis in BAT is of special interest here as it undergoes a marked morphological and functional adaptation after chronic cold exposure. Consequently, activation of BAT enhances glucose and lipid uptake and oxygen consumption due to increased metabolic demand to support thermogenesis and energy expenditure [8, 10]. Specifically for ATP synthase, chronic activation of BAT by exposure to cold results in an increase in the reverse mode of ATP synthase at the expense of ATP hydrolysis, but not in the forward mode (i.e., ATP synthesis). This is associated with downregulation of IF1 levels [13, 33] (Figure 2). In this context, it is important to consider the ratio of IF1/ATP synthase protein levels or activity, which is more relevant for interpreting the biological implications of IF1^29^. In BAT, a third component needs to be added to this scenario: UCP1, which is stimulated in activity and expression by cold exposure [35]. Embedded in the inner mitochondrial membrane, UCP1 activation by fatty acids and removal of nucleotides increases proton conductance [9, 35], lowers MMP, increases mitochondrial respiration, and generates heat [1, 4]. To provide a framework for the scenario, we and others have shown that ATP synthase hydrolytic activity is increased in BAT upon chronic cold exposure without modulation of ATP synthase protein levels [13]. At first, it could be hypothesized that increased UCP1 proton conductance would be enough to drive ATP synthase into its reverse mode. However, the observation of intact, albeit low, ATP synthesis in cold‐adapted BAT alongside marked downregulation of IF1 protein levels suggests that greater UCP1 activity alone is insufficient to elevate ATP synthase hydrolytic activity [13]. Low ATP synthesis [34] and the response to oligomycin [20] suggest substantial rates of coupled respiration in activated BAT when UCP1 is active. In fact, creatine‐ and lipid‐driven futile cycles that rely on coupled respiration as a source of ATP have been shown to contribute to NST [36, 37]. Considering the heterogeneity of mitochondrial populations in thermogenic adipose tissue, future work should reconcile the contribution of coupled respiration, ATP synthase hydrolytic activity, and UCP1‐mediated uncoupled respiration for NST.

Given the positive net balance of ATP hydrolytic activity on MMP, the greater ATP synthase hydrolytic activity in the BAT of cold‐adapted mice may seem counterintuitive to UCP1‐dependent thermogenesis at first glance. However, in vitro and in vivo overexpression of IF1 in BAT reduces mitochondrial respiration and adrenergic‐induced uncoupling response in cold‐adapted mice, suggesting that the downregulation of IF1 in BAT exerts not only an effect on ATP synthase hydrolytic activity but also on overall mitochondrial uncoupling capacity upon adrenergic stimulation. As raising temperature from (22°C) to thermoneutrality (30°C for mice) does not modulate IF1, and IF1 whole‐body deficiency is insufficient to modulate adrenergic response in BAT, even at thermoneutrality downregulation of IF1 in BAT seems to represent an intrinsic phenomenon specifically linked and necessary for the broader adaptation of BAT to marked cold exposure [13]. Unlike oxygen consumption, the relationship between IF1 and NST does not appear to be linear, and there might be a certain temperature threshold below which IF1 needs to be removed from the system to allow proper NST.

## 3 IF1 Beyond Thermogenesis in Brown Adipocytes

BAT undergoes extensive remodeling to adapt to cold [1, 38]. Among these processes are inhibition of apoptosis and induction of preadipocyte proliferation, which are both found early during BAT recruitment upon cold exposure [40]. Mitochondrial depolarization is a key trigger for apoptosis by triggering the release of pro‐apoptotic factors (i.e., cytochrome c) [41]. Therefore, a critical question is how mitochondria maintain MMP when UCP1 conductance is high without risking triggering apoptosis. In mitochondria overexpressing IF1, adrenergic mitochondrial depolarization is heightened [13]. Although no quantitative measurements of membrane potential voltage were performed in these experiments, it seems plausible that the downregulation of IF1 following cold exposure in mice seems to be a protective mechanism to sustain MMP and avoid apoptosis. In the future, it would be interesting to determine how IF1 modulation affects apoptosis rate in brown adipocytes as well as cell fate during cold‐induced recruitment of new adipocytes. In addition, in HEK293T cells, manipulation of IF1 levels and MMP modulates DNA methylation levels [42], which was associated with the specific regulation of lipid and carbohydrate metabolism‐related genes. Therefore, IF1 downregulation in BAT following cold exposure could also affect epigenetic regulation, although the mechanism through which MMP affects DNA methylation still needs to be defined.

Mitochondrial morphology is linked to substrate utilization in various contexts and cells/tissues [42, 43, 44]. When physically bound to ATP synthase, IF1 promotes ATP synthase dimerization, influencing the shape of the mitochondrial cristae [45, 46, 47]. Cristae shape is considered to alter ETC efficiency to generate the electrochemical gradient [49], therefore, directly affecting OxPhos and thermogenesis [50]. By applying transmission electron microscopy integrated with confocal fluorescence, it would be possible to co‐localize IF1 and UCP1 within mitochondria during the morphological adaptation BAT undergoes during cold exposure. In addition, IF1 manipulation modulates mitochondrial dynamics and cell function in various scenarios [50, 51]. For example, compared to fused mitochondria, fragmented ones have a greater capacity to utilize lipids due to lower malonyl‐CoA sensitivity [53]. Importantly, mitochondria associated with lipid droplets in BAT retain their ability to generate ATP by utilizing lipids from lipid droplets [18]. Although IF1 distribution in these two (or more) mitochondrial subpopulations is still unknown, silencing of IF1 in brown adipocytes favors mitochondrial lipid oxidation to sustain thermogenesis despite greater cytosolic glycolysis [13]. Therefore, subcellular fractionation of mitochondrial populations upon labeled substrate tracing could inform how manipulation of IF1 levels in brown adipocytes rewires metabolism. In HeLa cells, IF1 overexpression reduces NADH levels [48], which can modulate activity of several intra‐mitochondrial metabolic pathways, including carbohydrate metabolism (i.e., pyruvate dehydrogenase), lipid metabolism (i.e., 3‐hydroxy acyl‐CoA dehydrogenase), and tricarboxylic acid cycle (i.e., isocitrate dehydrogenase), among others. Altogether, it is reasonable to speculate that the ability of IF1 to influence substrate utilization involves changes in mitochondrial morphology exerted by dimerization of ATP synthase. However, empirical evidence to support this hypothesis in UCP1^+^ brown adipocytes is still lacking. It is noteworthy that IF1 levels in white adipocytes are almost undetectable (data not shown), and therefore, given the known downregulation of IF1 following cold exposure in BAT, it is unlikely that IF1 has any role in cold‐induced beigeing of white adipocytes. To address how IF1 modulation influences mitochondrial metabolism and substrate selection beyond thermogenesis in BAT, a comprehensive evaluation of mitochondrial morphology as well as metabolic tracing with stable isotopes, is necessary. These techniques are sensitive enough to pinpoint underappreciated rewiring of metabolism, which oxygen consumption alone cannot distinguish.

## 4 Conclusions

**FIGURE 3:**
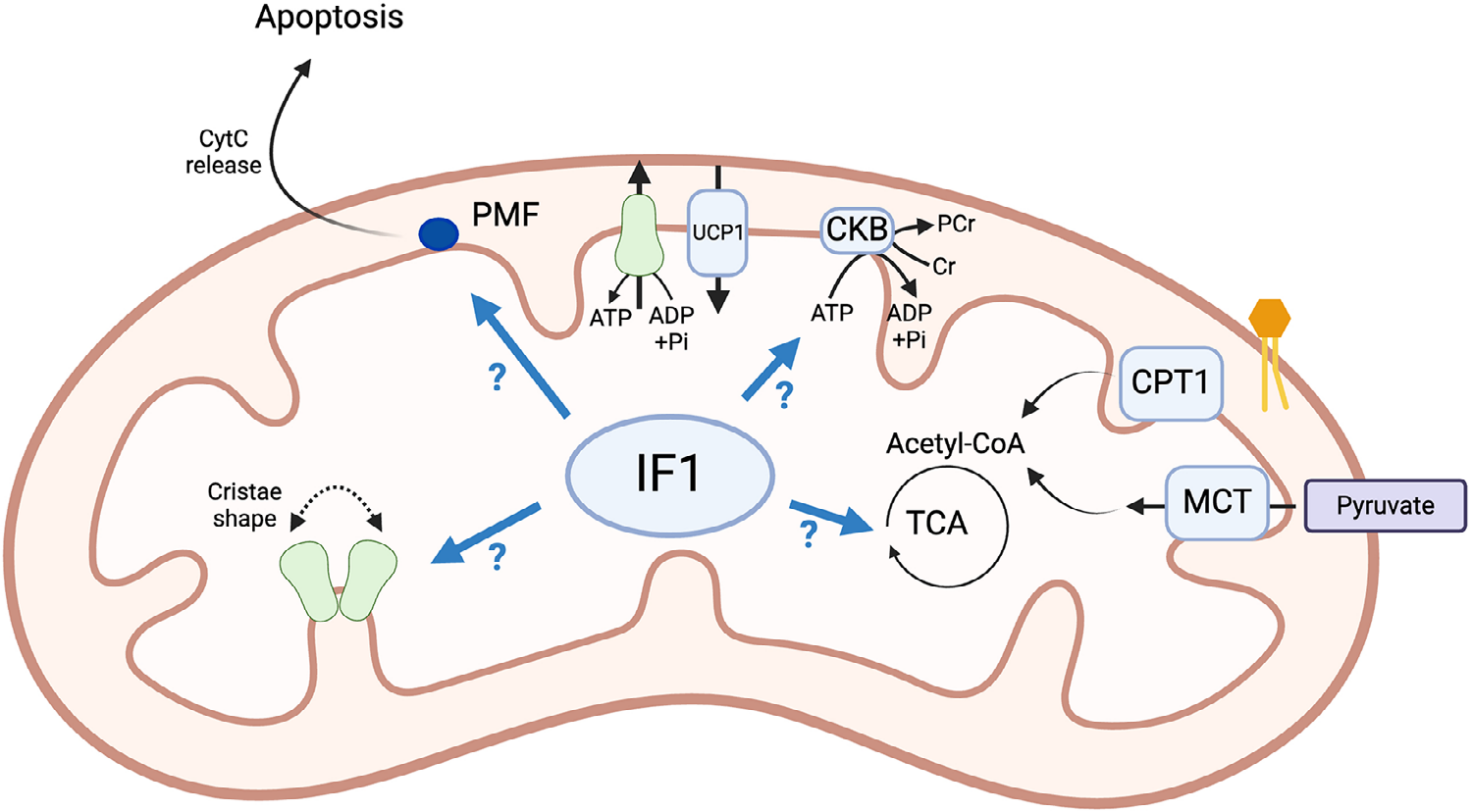
Possible implications of IF1 modulation on brown adipocytes biology. IF1 biology seems to be broader than previously hypothesized. Here, we highlight unanswered questions regarding the role of IF1 in BAT biology.

The metabolic challenge imposed by cold exposure reshapes BAT morphology and function. Maintenance of MMP due to greater hydrolytic activity of ATP synthase, alongside downregulation of its endogenous regulator IF1, is necessary for the full activation of BAT upon adrenergic signaling. Work from non‐thermogenic cells suggests that IF1 modulation impacts not only the activity of ATP synthase but also mitochondrial morphology, cristae shape, overall metabolism, and cell fate (Figure 3); however, whether IF1 participates in these processes during BAT remodelling following cold exposure is yet to be determined.

## Author Contributions

Henver Brunetta wrote the manuscript with input from Marcelo A. Mori and Alexander Bartelt. All authors approved the final manuscript.

## Conflicts of Interest

The authors declare no conflict of interest related to this work.

## Data Availability

Data sharing is not applicable to this article as no datasets were generated or analyzed during the current study.
